# Evaluation of myocardial classic and alternative pathways of the renin-angiotensin system in cholestasis-induced cardiac injury: A time-course experimental study in rats

**DOI:** 10.22038/ijbms.2025.87090.18818

**Published:** 2025

**Authors:** Azadeh Khalili, Parham Samimisedeh, Mohammad Maleki, Elmira Jafari Afshar, Mohammad Jadidian, Roham Mazloom, Gholamreza Bayat, Hossein Karim

**Affiliations:** 1 Department of Physiology-Pharmacology-Medical Physics, School of Medicine, Alborz University of Medical Sciences, Karaj, Iran; 2 Cardiovascular Research Center, Alborz University of Medical Sciences, Karaj, Iran; 3 Department of Pathology, School of Medicine, Alborz University of Medical Sciences, Karaj, Iran; 4 Department of Cardiology, School of Medicine, Alborz University of Medical Sciences, Karaj, Iran

**Keywords:** Angiotensin-converting - enzyme 2, Angiotensin receptors, Cholecardia syndrome, Cholestasis, Renin-angiotensin system

## Abstract

**Objective(s):**

Cholestasis, characterized by impaired bile flow and elevated bile acid levels, can lead to cardiac dysfunction, termed Cholecardia syndrome. The pathophysiology of cholestasis-induced cardiac injury involves direct and indirect effects of bile acids through various molecular pathways. However, the role of the renin-angiotensin-aldosterone system (RAS), modulated by bile acids, remains unclear.This study aimed to investigate alterations in the expression of classic and alternative RAS components in the myocardium of rats with obstructive cholestasis in a six-week period.

**Materials and Methods:**

Forty-two male Wistar rats (8 weeks old, 250 ± 30 g) were randomly assigned to seven groups (n=6 per group): one sham-operated group and six bile duct ligation (BDL) groups, sacrificed at weekly intervals from 1 to 6 weeks post-surgery. Quantitative RT-PCR was used to analyze cardiac RAS component expression. Biochemical and histopathological evaluations were conducted to assess disease progression.

**Results:**

In the classic RAS pathway, myocardial angiotensin-converting enzyme (ACE) expression increased after week 4 of BDL, while angiotensin II type 1 receptor (AT1R) and angiotensin II type 2 receptor (AT2R) were significantly down-regulated in this period. In contrast, the alternative RAS pathway components, including ACE2 and the Mas receptor, exhibited a biphasic expression pattern in the myocardium, with down-regulation at week 3 followed by significant up-regulation at weeks 5–6.

**Conclusion:**

The findings reveal distinct alterations in RAS pathways during cholestasis-induced cardiac injury. The alternative RAS pathway may play a compensatory role in late-stage cholestasis, highlighting potential therapeutic targets for Cholecardia syndrome and cirrhotic cardiomyopathy.

## Introduction

Liver cholestasis, marked by the disruption of bile flow and subsequent accumulation of bile acids in the liver and circulation (1), is associated with hepatic injury and various extrahepatic manifestations (2, 3). Among these, cardiac dysfunction emerges as a particularly significant clinical concern (4). Cardiac impairment can arise in both acute and chronic cholestasis. In acute cholestasis, it may be attributed to the detrimental impact of excess bile acids on the myocardium (5). Meanwhile, chronic cholestasis can progress to liver cirrhosis, which can result in cirrhotic cardiomyopathy, a complex condition characterized by both myocardial diastolic and systolic dysfunction (6). The term “Cholecardia syndrome” describes cardiac dysfunction attributed to liver cholestasis. This syndrome encompasses a spectrum of manifestations, including cardiac arrhythmias, aberrant heart rate variability, myocardial hypertrophy, myocardial remodeling, and fibrosis, culminating in heart failure (5, 7). While the precise mechanism underlying Cholecardia syndrome remains unclear, numerous studies have highlighted elevated bile acid levels as a prominent factor in its pathophysiology (5, 8, 9). Abnormally high levels of bile acids can induce cardiotoxicity mainly through mechanisms such as activation of the caspase pathway, resulting in myocyte apoptosis, myocardial injury, and remodeling (10), disturbances in calcium ion homeostasis (11), and impairment in heart fatty acid oxidation (10, 11).

Previous studies have revealed a down-regulation of Peroxisome Proliferator-activated Receptor-γ Coactivator 1-α (PGC1α), a key regulator of fatty acid oxidation in the heart, in obstructive cholestasis induced by bile-duct ligation (BDL) models in rats, whereas such down-regulation was not observed in non-cholestatic liver fibrosis, emphasizing the possible direct adverse impact of cholestasis on cardiac function (5, 12). The renin-angiotensin system (RAS) plays a crucial role in cardiovascular physiology and also contributes to various pathophysiological conditions, including myocardial remodeling (13), fibrosis (14), and heart failure (15). It consists of two pathways, the classic and alternative, which exert opposing effects (16). The detrimental impact of the classic RAS pathway mediated by the Angiotensin Converting Enzyme(ACE)/ angiotensin II/Angiotensin II Type 1 Receptor (AT1R), alongside the protective effects of the counterregulatory pathway involving ACE2/Angiotensin 1-7 (Ang 1-7)/Mas receptor (MasR) axis, in liver fibrosis resulting from cholestatic liver disease, has been well-documented (17). However, their involvement in Cholecardia syndrome remains inadequately elucidated. Hence, the primary objective of our present study is to unveil any changes in the components of the alternative RAS pathway—a more recently discovered arm of the RAS—within the myocardium of rats with obstructive cholestasis. Additionally, we aim to assess changes in the expression of classic RAS components in the myocardium to enhance understanding and facilitate comparison. By shedding light on the alteration patterns in alternative and classic RAS components during both subacute and chronic obstructive cholestasis in this present time-course study, our research serves a dual purpose. Firstly, it enhances our comprehension of the underlying pathophysiology implicated in Cholecardia syndrome, leveraging a six-week evaluation period to assess key components in detail. Secondly, it provides valuable groundwork for subsequent investigations aimed at evaluating the therapeutic potential of targeting the alternative RAS pathway for the prevention and treatment of Cholecardia syndrome and cirrhotic cardiomyopathy.

## Materials and Methods

### Animals

Forty-two male Wistar rats, aged eight weeks and weighing 250 ± 30 grams, were selected for the experimental investigation. These rats were housed under standard conditions, with a 12-hour light/dark cycle, controlled room temperature (maintained at 22 ± 2 °C), and relative humidity of 50 ± 10%. Prior to the commencement of experiments, the rats underwent a minimum one-week acclimatization period to these environmental conditions, during which they had access to food and water ad libitum. The research adhered to guidelines regulating the care and utilization of experimental animals (18). Approval for the study was secured from the research ethics committee at Alborz University of Medical Sciences (IR.ABZUMS.REC.1398.159). 

### Experimental design

The rats were distributed randomly into seven groups, each consisting of six animals. One group was designated as the surgical sham-operated group, while the remaining experimental groups comprised rats undergoing bile-duct ligation (BDL) for varying durations: 1-week BDL (BDL-1W), 2-weeks BDL (BDL-2W), 3-weeks BDL (BDL-3W), 4-weeks BDL (BDL-4W), 5-weeks BDL (BDL-5W), and 6-weeks BDL (BDL-6W). All rats in the sham-operated and experimental groups, regardless of group assignment, underwent the surgical procedure. Following this, each group was sacrificed at one-week intervals over a total period of six weeks.

### Experimental protocols

*Surgical obstructive cholestasis protocol*

The bile duct ligation procedure followed the method described by Yang *et al*. (19). Briefly, the bile duct ligation surgery was conducted under aseptic conditions. Anesthesia was induced by intraperitoneal injection of a solution containing ketamine (60 mg/kg) and Xylazine (8 mg/kg) (Alfasan, Woerden, Netherlands) **(**20**).** Once the rats exhibited diminished reflexes, they were securely positioned on a thermal pad adjusted at 37 °C. A vertical incision of approximately 1-1.5 cm was made along the upper abdomen midline to access the bile duct. Careful dissection was performed to expose and isolate the common bile duct from surrounding tissues. Using 4-0 silk sutures, the bile duct was ligated twice near the junction of the hepatic ducts and just above the entry point of the pancreatic duct. Subsequently, the common bile duct between the two ligatures was cut to prevent recanalization. Following ligation, the abdominal wall and fascia were sutured with continuous 4-0 catgut sutures, while the skin was closed using simple interrupted 4-0 nylon sutures. Tetracycline ointment was applied post-surgery to prevent probable infection and suture chewing. In the sham surgical group, the bile duct was exposed without ligation following the upper midline incision. Following surgery, rats were individually housed in separate cages and closely monitored for any signs of morbidity or mortality. To ensure adequate postoperative analgesia, all rats received buprenorphine (0.05 mg/kg, subcutaneously) every 12 hr for three consecutive days following BDL surgery.

*Biochemical analyses*

At the time of sacrifice, each group of animals received deep anesthesia via intraperitoneal injections of ketamine (80 mg/kg) and Xylazine (8 mg/kg), followed by a thoracotomy procedure. Approximately 5 cc blood samples were gently collected from the right ventricle of the heart. Following collection, the samples were promptly centrifuged, and the plasma was stored at 4 °C. Hepatic markers, such as aspartate aminotransferase (AST), alanine aminotransferase (ALT), alkaline phosphatase (ALP), gamma-glutamyl transferase (GGT), albumin, and total and direct bilirubin, were assessed using commercially available kits as per the manufacturer’s guidelines. Biochemical analyses for the control and surgical-sham groups were conducted upon completion of the study.

### Histopathology

*Heart sample preparation for histopathology techniques*

After sacrifice, the animals’ hearts were dissected and weighed, and a portion of the left ventricle was immediately excised and fixed in a 10% formalin solution. The cardiac tissue samples were then analyzed using hematoxylin and eosin (H&E) staining.

*Quantitative RT-PCR technique*

To accurately assess the expression levels of target genes, which encompass components of alternative RAS pathways such as ACE2, Ang 1-7 receptor (MasR), as well as classic RAS pathway components including Angiotensin Type 1 Receptor (AT1R), Angiotensin Type 2 Receptor (AT2R), ACE1, and Transforming Growth Factor-beta (TGF-beta) quantitative real-time reverse transcription polymerase chain reaction (RT-PCR) was conducted following a standardized protocol. In brief, approximately 30 mg of left ventricle tissue was homogenized using a Polytron tissue homogenizer (DAIHAN-brand Homogenizing Stirrer, HS-30E; Korea). Total RNA extraction was performed using the RNeasy Fibrous Tissue Mini Kit (Yekta Tajhiz Azma Co., Tehran, Iran), following the manufacturer’s guidelines. RNA concentration and quality were assessed by measuring the absorbance ratio at 260/280 nm, with an acceptable range defined as 1.9 to 2.2. The Yekta Tajhiz Azma First Strand cDNA Synthesis Kit was employed for cDNA synthesis, adhering to the manufacturer’s instructions. Real-time PCR was conducted using SYBR green as the fluorescent dye (Yekta Tajhiz Azma Co., Iran). To ensure accuracy and reliability, experiments were performed in duplicate, following a specific protocol (21). The protocol commenced with a preincubation step at 95 °C for ten minutes, followed by 45 cycles of amplification. Each cycle comprised denaturation at 95 °C for 10 sec, annealing at 60 °C for 10 sec, and extension at 72 °C for 10 sec. Subsequently, the melting stage included steps at 95 °C, 65 °C, and 97 °C for 10 sec, 60 sec, and 60 sec, respectively. Expression levels were normalized to the housekeeping gene GAPDH and presented as fold change (FC) compared to the sham-operated group (Table 1).

### Data and statistical analyses

Except for the histological findings, the data were expressed as mean ± SEM. Between-group comparisons were conducted using One-way analysis of variance (ANOVA). If a significant difference was observed, Duncan’s multiple range test was employed as a post hoc analysis to determine specific differences among the means. Histological data were analyzed using the Kruskal-Wallis nonparametric test and presented as median values with interquartile ranges (IQR). Statistical significance was defined as a P-value less than 0.05. Graphs were created using GraphPad Prism version 10.3.0 (GraphPad Software; San Diego, California, USA).

## Results

### Absolute heart weights and heart weights to body weight ratio

The absolute heart weights, along with their respective ratios to the rats’ final body weights, are summarized in Table 2.

The absolute heart weights ranged from 790.4 ± 22 mg in BDL-1W to 1058 ± 60 mg in BDL-6W. While heart weights from BDL-1W to BDL-5W were generally lower than those in the sham-operated group, the sole significant difference was observed for BDL-1W (*P*<0.01). Moreover, the mean absolute heart weights in the BDL-6W group were significantly higher than those in BDL-1W (*P*<0.01), BDL-2W (*P*<0.05), BDL-3W (*P*<0.05), and BDL-4W (*P*<0.05).

Regarding the heart weight to total body weight ratio, although there was an observed increase in the relative heart weights in BDL-1W and BDL-2W compared to the sham-operated group, these differences were statistically insignificant. Conversely, statistically significant increases were observed in the BDL-3W to BDL-6W groups compared to the sham-operated group (*P*<0.05 for all comparisons). Moreover, no significant differences were found among the experimental groups.

### Biochemical analysis

Liver enzyme measurements, including AST, ALT, and ALP, as well as total and direct bilirubin, revealed statistically significant increases across all experimental groups (BDL-1W to BDL-6W) compared to the sham-operated group. Specifically, AST levels were higher in BDL-1W to BDL-6W than in the sham-operated group (*P*<0.001 for all comparisons) with an upward trend from BDL-1W to BDL-5W. The between-group comparison revealed that, compared to the BDL-1W, AST levels were significantly higher in BDL-2W (*P*<0.05), BDL-3W (*P*<0.01), BDL-4W (*P*<0.01), BDL-5W (*P*<0.001), and BDL-6W (*P*<0.001). Moreover, AST levels were significantly higher in BDL-4W (*P*<0.05), BDL-5W (*P*<0.01), and BDL-6W (*P*<0.01) compared to BDL-2W. Additionally, BDL-5W and BDL-6W exhibited significantly elevated AST levels compared to BDL-3W (*P*<0.01 for both comparisons).

Similar to AST, ALT levels were also significantly elevated in all experimental groups compared to the sham-operated group (*P*<0.01, *P*<0.001, *P*<0.001, *P*<0.001, *P*<0.001, *P*<0.001, for BDL-1W to BDL-6W, respectively). Among the experimental groups, ALT levels were highest in BDL-6W, showing a significant increase compared to BDL-1W (*P*<0.05). No other significant differences were observed between the remaining groups.

 Additionally, ALP levels were significantly elevated in all BDL-1W to BDL-6W groups compared to the sham-operated group (*P*<0.001 for each). However, no significant differences in ALP levels were observed among the experimental groups.

Except for the BDL-1W group, all other groups exhibited significantly higher GGT levels compared to the sham-operated group (*P*<0.001 for all comparisons). Interestingly, when compared to the BDL-1W group, the GGT levels were significantly higher in the BDL-2W (*P*<0.001), BDL-3W (*P*<0.001), and BDL-4W (*P*<0.001) groups. Additionally, the GGT levels in the BDL-3W group were significantly higher than those in the BDL-2W (*P*<0.001), BDL-4W (*P*<0.001), BDL-5W (*P*<0.001), and BDL-6W (*P*<0.001) groups. The GGT levels in the BDL-4W group were also significantly higher compared to the BDL-2W (*P*<0.001)

Overall, albumin levels were significantly lower in all experimental groups compared to the sham-operated group, with the decreases being statistically significant in BDL-4W (*P*<0.001), BDL-5W (*P*<0.001), and BDL-6W (*P*<0.001). Additionally, the albumin levels in BDL-4W and BDL-5W were significantly lower than in BDL-3W (*P*<0.01 for both comparisons).

Furthermore, total and direct bilirubin levels were significantly elevated in all experimental groups compared to the sham-operated group (*P*<0.001 for all comparisons), with no significant differences among the experimental groups **(**Table 3).

### Real-time PCR for quantitative gene expression analysis

#### Alternative RAS components

*Angiotensin converting enzyme 2 (ACE2)*

**Figure 1:**
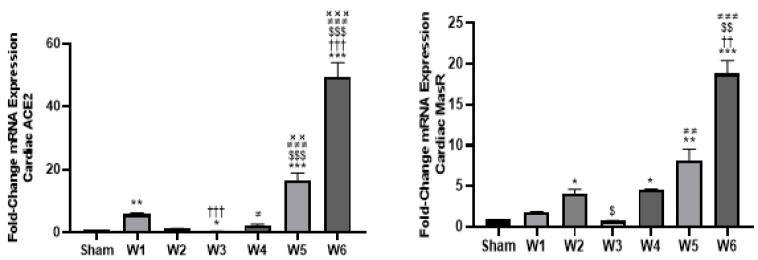
Dynamic myocardial expressions of alternative RAS components

The analysis of ACE2 mRNA expression in rat cardiac tissues revealed significantly elevated levels in BDL-1W (FC = 5.77, *P*<0.01), BDL-5W (FC = 16.4, *P*<0.001), and BDL-6W (FC = 49.4, *P*<0.001) compared to the sham-operated controls. In contrast, BDL-3W exhibited a significantly lower expression level (FC = 0.29, *P*<0.05). No significant differences were observed in BDL-2W and BDL-4W relative to the sham-operated group. In between-group comparisons, compared to BDL-1W, BDL-3W showed lower ACE2 expression (*P*<0.001), while BDL-6W exhibited significantly higher expression (*P*<0.001). Compared to BDL-2W, BDL-5W and BDL-6W had significantly higher myocardial ACE2 expression (*P*<0.001). Additionally, compared to BDL-3W, expression was significantly elevated in BDL-4W (*P*<0.05), BDL-5W (*P*<0.001), and BDL-6W (*P*<0.001). Finally, ACE2 expression in BDL-5W (*P*<0.01) and BDL-6W (*P*<0.001) was higher than in BDL-4W (Figure 1A).

*Ang 1-7 receptor (Mas receptor) mRNA*

Our time-course study elucidated a biphasic pattern in MasR mRNA expression over the study duration in rats’ cardiac tissues. Notably, the lowest average cardiac MasR expression was observed at BDL-3W, followed by a progressive increase leading up to BDL-6W. In this regard, compared to the sham-operated group, rats in BDL-2W (FC = 4.1, *P*<0.05) exhibited higher cardiac MasR mRNA expression, while there was no significant difference between BDL-1W and BDL-3W and the sham-operated group. Notably, progressively elevated expression levels of myocardial MasR were observed in BDL-4W (FC = 4.52, *P*<0.05), BDL-5W (FC = 8.2, *P*<0.01), and BDL-6W (FC = 18.8, *P*<0.001) compared to the sham-operated group. In between-group comparisons, significant differences in MasR expression were observed in BDL-6W compared to both BDL-1W (*P*<0.01), BDL-2W (*P*<0.01), and BDL-3W (*P*<0.001). Additionally, MasR expression in BDL-2W (*P*<0.05) and BDL-5W (*P*<0.01) was significantly higher than in BDL-3W. There was no other significant difference between groups (Figure 1B).

### Classic RAS components

*Angiotensin Converting Enzyme (ACE)*

**Figure 2:**
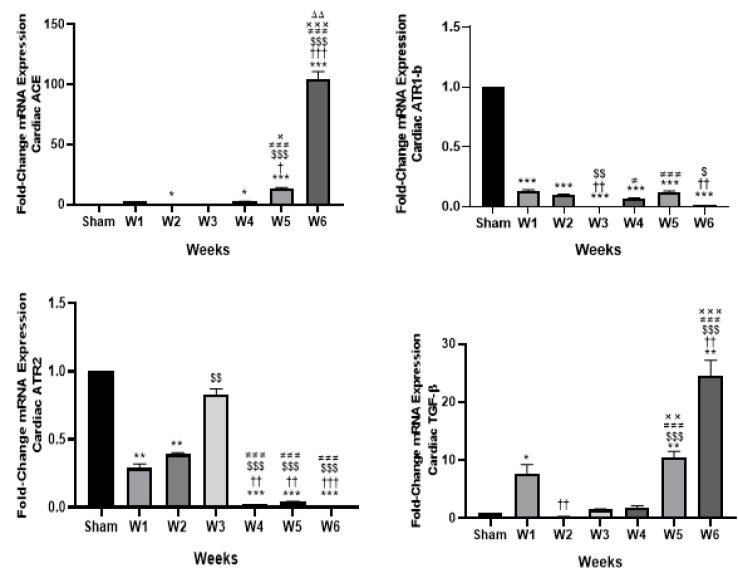
Dynamic myocardial expressions of the classic RAS components

Compared to the sham-operated group, only BDL-2W exhibited a significantly lower myocardial ACE1 expression (FC = 0.23, *P*<0.05). In contrast, the expression in BDL-4W (FC = 2.5, *P*<0.05), BDL-5W (FC = 13.1, *P*<0.001), and BDL-6W (FC = 104.4, *P*<0.001) was higher than that of the sham-operated group. Regarding between-group comparisons, BDL-5W showed significantly higher myocardial ACE expressions compared to the BDL-1W (*P*<0.05), BDL-2W (*P*<0.001), BDL-3W (*P*<0.001), and BDL-4W (*P*<0.05). The highest myocardial ACE expressions were observed six weeks after BDL, which were significantly higher compared to the BDL-1W (*P*<0.001), BDL-2W (*P*<0.001), BDL-3W (*P*<0.001), BDL-4W (*P*<0.001), and BDL-5W (*P*<0.01) (Figure 2A).

*Angiotensin type 1 receptor (AT1R)*

We also investigated the myocardial expression changes of AT1R-b and observed an overall decrease in gene expression in the experimental group compared to the sham-operated group.

Myocardial AT1R-b mRNA expression, were significantly lower in BDL-1W (FC = 0.13), BDL-2W (FC = 0.1), BDL-3W (FC = 0.008), BDL-4W (FC = 0.07), BDL-5W (FC = 0.12), and BDL-6W (FC = 0.01) compared to the sham-operated group (*P*<0.001 for all comparisons). The lowest AT1R myocardial expressions were observed in BDL-3W, which were significantly lower compared to the BDL-1W (*P*<0.01), BDL-2W (*P*<0.01), BDL-4W (*P*<0.05), and BDL-6W (*P*<0.01). The BDL-6W group also demonstrated significantly lower AT1R-b mRNA expression compared to the BDL-1W (*P*<0.01) and BDL-2W (*P*<0.05) **(**Figure 2B).

### Angiotensin type 2 receptor (AT2R)

The myocardial expression of AT2R, similar to that of AT1R, was reduced in all experimental groups compared to the sham-operated group. This reduction was statistically significant in all groups except for BDL-3W (*P*<0.01, <0.01, <0.001, <0.001, and <0.001 for BDL-1W through BDL-6W, respectively). Compared to the BDL-1W group, BDL-4W (*P*<0.01), BDL-5W (*P*<0.01), and BDL-6W (*P*<0.001) exhibited significantly lower myocardial AT2R expression. Similarly, when compared to BDL-2W, BDL-4W to BDL-6W showed significantly lower myocardial AT2R expressions (*P*<0.001 for all comparisons); in contrast, BDL-3W demonstrated significantly higher AT2R expression compared to BDL-2W (*P*<0.01). Furthermore, myocardial AT2R expression in BDL-4W to BDL-6W was reduced considerably compared to the BDL-3W group (*P*<0.001 for all comparisons) (Figure 2C).

### Transforming growth factor (TGF) beta

Except for BDL-2W, which exhibited lower myocardial TGF-beta expression compared to the sham-operated group, all other time points demonstrated increased expression levels. The differences were statistically significant for BDL-1W (FC = 7.74, *P*<0.05), BDL-5W (FC = 10.5, *P*<0.01), and BDL-6W (FC = 24.6, *P*<0.01) compared to the sham-operated group. Additionally, myocardial TGF-beta expression in BDL-2W was significantly lower than in BDL-1W (*P*<0.01), whereas BDL-6W showed substantially higher expression compared to BDL-1W (*P*<0.01). Both BDL-5W and BDL-6W exhibited significantly increased myocardial TGF-β expression compared to BDL-2W (*P*<0.001 for both), BDL-3W (*P*<0.001 for both), and BDL-4W (*P*<0.01 vs BDL-5W; *P*<0.001 vs BDL-6W) (Figure 2D).

### Histopathology

*Heart histopathology*

**Figure 3:**
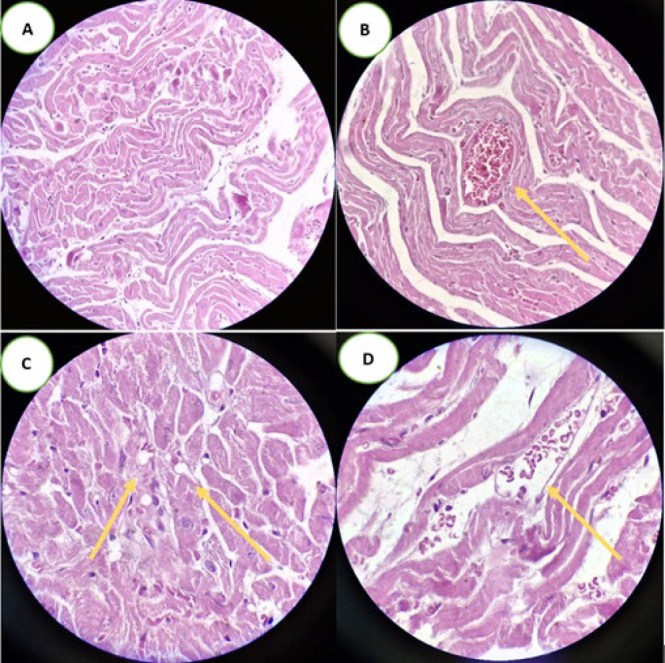
Light photomicrographs of histological sections of the rat left myocardium stained with H&E (magnification ×400)

We evaluated the heart samples using H&E staining with following indices: Cardiomyocyte hypertrophy, altered pigmentation, myofiber Vacuolization, interstitial fibrosis, disorganized cardiac muscle fibers (separated muscle fibers), increased number of fibroblasts between the cardiac muscle fibers, cytoplasmic lysis, congested blood capillaries, extravasated red blood cells, cell infiltration, eosinophilic changes, contraction band necrosis, enlargement and internalization of nuclei, loss of nuclei, pyknosis, and karyolysis. As anticipated, only minor changes were observed in cardiac histopathology across the groups. Compared to the sham-operated group, the BDL-5W group exhibited mild disorganization of cardiac muscle fibers and the presence of extravasated red blood cells **(**Figure 3). These differences were statistically significant, with *P*-values of <0.001 and <0.01, respectively. No other statistically significant differences were observed between the groups. A detailed comparison between groups is presented in Table 4.

## Discussion

In this longitudinal investigation conducted on bile duct ligated rats, we observed a dynamic modulation in the expression of components within the RAS. Specifically, we examined alterations in both alternative pathways, namely ACE2 and the Angiotensin 1-7 receptor (Mas receptor), as well as the classic pathway, including ACE, AT1R, and AT2R, within myocardial tissue over time. Our study revealed a bimodal pattern in the expression of Mas receptor and ACE2 mRNAs, with a significant up-regulation observed during the progression of cholestatic-induced liver cirrhosis beyond the third week, post-bile duct ligation. However, at week three post-bile duct ligation, a considerable down-regulation was evident. This delayed surge in the expression of alternative RAS components is believed to represent a compensatory mechanism aimed at mitigating the unfavorable effects of Ang II on the myocardium (22). Additionally, histopathological analysis demonstrated only mild changes in cardiac tissue across experimental groups. The most notable alterations, observed in the BDL-W5 group, included mild disorganization of cardiac muscle fibers and extravasated red blood cells, which were statistically significant compared to the sham-operated group. Biochemical analyses confirmed progressive liver dysfunction, as indicated by significant elevations in liver enzymes (AST, ALT, and ALP), bilirubin levels, and GGT, along with decreased albumin levels in later stages of cholestasis. Previous experimental and clinical studies have consistently documented elevated levels of circulating Angiotensin I and Angiotensin II following cholestasis and liver cirrhosis, which have been implicated in the pathogenesis of myocardial fibrosis, ventricular remodeling, and cardiac dysfunction (23-25). ACE2, by catalyzing the degradation of Angiotensin II to Angiotensin 1-7, is assumed to counteract the detrimental effects of Angiotensin II on myocardial function (26). Notably, our study revealed a delayed activation of this compensatory mechanism, consistent with observations reported by Österreicher *et al*. in their investigation of the alternative RAS pathway in the livers of bile duct-ligated rats. They documented a significant elevation in ACE and Angiotensin II during the acute phase of cholestatic-induced liver injury, followed by a gradual increase in alternative RAS components during the progression of chronic liver damage (27). The investigation of the ACE2/Ang 1-7/MasR axis as a potential therapeutic target in cardiovascular diseases, such as hypertension, has recently garnered significant interest in numerous research studies (28, 29). Alongside the endogenous agonist Ang 1-7, CGEN-856S and AVE0991 have been identified as novel Mas G-protein-coupled receptor agonists, exhibiting favorable effects in cardiovascular protection (30). In Savergnini *et al*.’s study, the administration of CGEN-856S to Wistar rats resulted in reduced infarct size in the setting of myocardial infarction, diminished collagen deposition in myocardial tissue, and attenuated cardiac remodeling induced by isoproterenol (31). Given the observed delayed compensatory response in the alternative RAS axis components within heart tissues during cholestasis, studies exploring the early administration of MasR agonists to mitigate the acute detrimental effects of angiotensin 2 on both hepatic and cardiac functions are warranted. The activation of the Mas receptor is implicated in the initiation of several signaling pathways, which enable it to exert its physiological effects. Concerning the PI3K/Akt/eNOS pathway, upon stimulation of the Mas receptor by Ang 1-7 or its agonists, downstream signaling cascades are initiated, resulting in the activation of Phosphoinositide 3-kinases (PI3K) (32). This activation subsequently leads to the phosphorylation of protein kinase Akt. Akt, in turn, phosphorylates endothelial nitric oxide synthase (eNOS), ultimately leading to the production of nitric oxide (33). The PI3K/Akt signaling pathway is involved in several physiological and pathological downstream cascades (34-36). Previous studies have reported that hydrophilic bile acids, such as ursodeoxycholic acid (UDCA), can exert cardioprotective effects via the PI3K/Akt-dependent pathway in ischemia-reperfusion injury (37). In contrast, abnormally elevated levels of hydrophobic bile acids during liver cholestasis may aberrantly redirect the PI3K/Akt signaling pathway to pathological downstream cascades, ultimately resulting in cardiac fibrosis and apoptosis (38). 

**Table 1:** Primer sequences of renin–angiotensin system (RAS) components used for real-time PCR analysis in Wistar rats

**Genes**	**Primary sequences (5** **′** ** - 3** **′** **)**	
	Forward	Reverse
**ACE**	CCTCTACCTGAACCTCCATG	GGCACTACCATGTCGTAAATG
**ACE2**	TGTTAGAGAAGTGGAGGTGG	GCAGGGTCACAGTATGTTTC
**ATR1**	CGTCTTGTTTTGAGGTGGAG	CGTAAGCCATTTAGTCAGAGG
**ATR2**	ATCTTCAATCTGGCTGTGG	GTCCAAAGAGCCAGTCATATC
**Mas Receptor**	CGGCTTTCTGGATTCTCAAAG	ATTCCCTTCCTGTTTCTCATG
**TGFβ**	CCTGAGTGGCTGTCTTTTG	GGACTGATCCCATTGATTTCC
**GAPDH (Housekeeping)**	GCCTTCTCTTGTGACAAAGTG	CTTCCCATTCTCAGCCTTG

PCR: Polymerase chain reaction, ACE: Angiotensin converting enzyme, ATR: Angiotensin receptor, TGFβ: Transforming growth factor β, GAPDH: Glyceraldehyde-3-phosphate dehydrogenase

**Table 2:** Absolute heart weights and heart-to-body weight in Sham-operated and bile duct ligation (BDL) experimental groups of Wistar rats (BDL-1W to BDL-6W)

Group/Variables	Total body weights (g)	Heart weights (mg)	Relative heart weights (mg/g)
Sham-operated	346.1 (50)	1024.1 (41)	2.0 (0.96)
BDL-1W	255.8 (20.9)	790.4 (22) ^**^	3.0 (0.11)
BDL-2W	284.1 (70.7)	897.2 (47)	3.0 (0.12)
BDL-3W	250.1 (20.8)	876.2 (52)	3.0 (0.53) ^*^
BDL-4W	249.1 (40.1)	862.6 (22)	3.0 (0.51) ^*^
BDL-5W	258.8 (90.2)	916.8 (30)	3.0 (0.55) ^*^
BDL-6W	291.1 (50.8)	1058.1 (60) ^††^^$^^#^^×^	3.0 (0.64) ^*^

*P<0.05, **P<0.01 compared to the Sham-operated group, ††*P*<0.01 compared to the BDL-1W, $*P*<0.05 compared to the BDL-2W, #*P*<0.05 compared to the BDL-3W, ×*P*<0.05 compared to the BDL-4W. Data are presented as mean ± SEM. BDL: Bile duct ligation, W: Week/ gr: gram, mg: Milligram

**Table 3:** Biochemical analysis of hepatocellular and functional liver enzymes in Sham-operated and bile duct ligation (BDL) experimental groups (BDL-1W to BDL-6W)

	**AST**	**ALT**	**ALP**	**GGT**	**Albumin**	**Direct bilirubin**	**Total bilirubin**
**Sham-operated**	121.3±9.0	97.0±6.17	367.8±26.3	4.5±0.56	3.83±0.08	0.13±0.02	0.50±0.03
**BDL-1W**	259.3±23.8^***^	182.7±22.7^**^	1034±79.0^***^	6.0±1.37	3.42±0.09	8.28±0.57^***^	8.75±0.60^***^
**BDL-2W**	407.3±57.5^***†^	201.0±20.4^***^	828.5±42.0^***^	17.0±2.5^***†††^	3.34±0.10	8.15±1.21^***^	9.08±1.32^***^
**BDL-3W**	504.3±31.25^***††^	252.2±21.3^***^	1046±73.0^***^	102.7±28.9^***†††^^$$$×××^	3.48±0.12	8.88±0.35^***^	9.57±0.35^***^
**BDL-4W**	569.0±36.2^***††^^$^	244.0±18.2^***^	1024±40.0^***^	27.3±4.60^***†††^^$$$^	2.86±0.11^***##^	7.60±0.36^***^	8.13±0.37^***^
**BDL-5W**	692.8±55.5^***†††^^$$^^##^	244.4±14.8^***^	938.3±106^***^	17.6±3.8^***###^	2.86±0.18^***##^	6.83±0.56^***^	7.56±0.60^***^
**BDL-6W**	674.8±42.1^***†††^^$$^^##^	252.5±48.0^***†^	910.0±73.3^***^	15.0±1.44^***###^	3.05±0.08^***^	7.00±0.76^***^	8.32±0.90^***^

**P<0.01, ***P<0.001 compared to the Sham-operated group, †*P*<0.05, ††*P*<0.01, †††*P*<0.001 compared to the BDL-1W , $*P*<0.05, $$*P*<0.01, $$$*P*<0.001 compared to the BDL-2W , ##*P*<0.01 compared to the BDL-3W , ×××*P*<0.001 compared to the BDL-4W .

W: Week, AST: Aspartate transaminase, ALT: Alanine transaminase, ALP: Alkaline phosphatase, GGT: Gamma-glutamyl transferase

Data are presented as Mean ± SEM

including Angiotensin Converting Enzyme 2 (ACE2) (A) and Mas Receptor (MasR) (B) in experimental groups, including Sham-operated and six bile duct ligated groups. Data is presented as mean ± SEM. *P<0.05, **P<0.01, ***P<0.001 compared to the Sham-operated group, ††*P*<0.01, †††*P*<0.001 compared to the BDL-1W $*P*<0.05, $$*P*<0.01, $$$*P*<0.001 compared to the BDL-2W , ≠*P*<0.05, ≠≠*P*<0.01, ≠≠≠*P*<0.001 compared to the BDL-3W, ××*P*<0.01, and ×××*P*<0.001 compared to the BDL-4W.

Including Angiotensin Converting Enzyme (ACE), Angiotensin Type 1 Receptor b (ATR1-b), and angiotensin type 2 receptor (ATR2) and transforming growth factor beta (TGF-B) in experimental groups, including Sham-operated and six bile duct ligated groups. Data is presented as mean ± SEM. *P<0.05, **P<0.01, ***P<0.001 compared to the Sham-operated group, †*P*<0.05, ††*P*<0.01, †††*P*<0.001 compared to the BDL-1W, $*P*<0.05, $$*P*<0.01, $$$*P*<0.001 compared to the BDL-2W, ≠*P*<0.05, ≠≠≠*P*<0.001 compared to the BDL-3W, ×*P*<0.05, ××*P*<0.01 and ×××*P*<0.001 compared to the BDL-4W, *P*<0.001 compared to BDL-5W.

**Table 4:** Histopathological analysis of cardiac tissue using Hematoxylin and Eosin (H&E) staining in Sham-operated and bile duct ligation (BDL) experimental groups of wistar rats (BDL-1W to BDL-6W)

	**Sham-Operated**	**BDL-1W **	**BDL-2W **	**BDL-3W **	**BDL-4W **	**BDL-5W **	**BDL-6W **
**Myofiber vacuolization**	1 (0, 1)	0 (0, 0)	1 (1, 1)	1 (0, 1)	0 (0, 0)	0 (0, 1)	0 (0, 0)
**Interstitial fibrosis**	0 (0, 0)	0 (0, 0)	0 (0, 0)	0 (0, 0)	1 (0, 1)	0 (0, 0)	0 (0, 0)
**Disorganized cardiac muscle fibers**	0 (0, 0)	0 (0, 0)	0 (0, 0)	0 (0, 0)	0 (0, 0)	1 (1, 1) ^***^	0 (0, 0)
**Congested blood capillaries**	0 (0, 1)	1 (0, 1)	1 (1, 1)	1 (1, 1)	1 (1, 1)	1 (1, 1)	1 (0, 1)
**Extravasated red blood cells**	0 (0, 0)	0 (0, 0)	0 (0, 0)	0 (0, 1)	1 (0, 1)	1 (1, 1) ^**^	0 (0, 1)
**Loss of nuclei**	0 (0, 0)	0 (0, 0)	0 (0, 0)	0 (0, 0)	0 (0, 0)	0 (0, 1)	0 (0, 0)
**Pyknosis**	0 (0, 0)	0 (0, 0)	0 (0, 0)	0 (0, 0)	0 (0, 0)	0 (0, 0)	0 (0, 0)
**Karyolysis**	0 (0, 0)	0 (0, 0)	0 (0, 0)	0 (0, 0)	0 (0, 0)	0 (0, 0)	0 (0, 0)

Histological changes were graded on a scale of 0 to 4, where 0 indicates no damage, 1 represents active damage involving less than 25% of the tissue, 2 corresponds to damage affecting less than 50%, 3 reflects damage extending to less than 75%, and 4 signifies active damage involving more than 75% of the tissue.

**P<0.01 ***P<0.001 vs Sham-operated group. The data are presented as median values with interquartile ranges (IQR). W: Week

Cardiomyocyte hypertrophy, altered pigmentation, increased fibroblast numbers, cytoplasmic lysis, cellular infiltration, eosinophilic changes, contraction band necrosis, nuclear enlargement and internalization, pyknosis, and karyolysis were also assessed across all groups; however, no significant changes were observed in any group.

(A) Disorganized cardiac muscle fibers; (B) Congested blood capillaries; (C) Myofiber vacuolization; (D) Extravasation of red blood cells.

## Conclusions

The expression of alternative renin-angiotensin system (RAS) components, including ACE2 and the Mas receptor, exhibited a biphasic pattern in rat hearts following bile duct ligation, with significant up-regulation observed after the third week. This observation suggests a potential delay in the counter-regulatory effects mediated by the alternative RAS axis in cholestasis-induced cardiomyopathy.

## Limitations and Future Perspectives

Our study has several limitations that should be acknowledged. First, the relatively short observation period may not fully capture the long-term effects of cholestasis on cardiac tissue, particularly the development of cirrhotic cardiomyopathy and its associated histopathological changes. A more extended follow-up period would provide a more comprehensive understanding of the progressive cardiac alterations induced by cholestasis.

Second, while we observed dynamic changes in RAS components, our study was limited to gene expression analysis. Future investigations incorporating protein-level validation and functional assays would strengthen our findings and provide deeper insights into the mechanistic role of RAS modulation in cholestasis-induced cardiac injury.

Additionally, our study did not evaluate the direct functional consequences of these molecular alterations on cardiac performance. Incorporating echocardiographic or hemodynamic assessments in future studies could further elucidate the impact of RAS modulation on cardiac function.

From a translational perspective, our findings lay the groundwork for future studies investigating potential therapeutic interventions. Evaluating the effects of pharmacological agents, particularly agonists of the alternative RAS pathway, at different stages of obstructive cholestasis may help determine their protective role in mitigating cholestasis-induced cardiomyopathy. Moreover, conducting comparative analyses using knockout models for alternative RAS components could provide more definitive evidence regarding their contribution to disease progression and potential therapeutic applications.

## Declaration

We have not used any AI tools or technologies to prepare this manuscript.
